# Smartphone-based particle image velocimetry for cardiovascular flows applications: A focus on coronary arteries

**DOI:** 10.3389/fbioe.2022.1011806

**Published:** 2022-12-08

**Authors:** Giuseppe C. A. Caridi, Elena Torta, Valentina Mazzi, Claudio Chiastra, Alberto L. Audenino, Umberto Morbiducci, Diego Gallo

**Affiliations:** PoliTo^BIO^Med Lab, Department of Mechanical and Aerospace Engineering, Politecnico di Torino, Turin, Italy

**Keywords:** Hemodynamics, PIV, stenosis, flow visualization, in vitro experiment

## Abstract

An experimental set-up is presented for the *in vitro* characterization of the fluid dynamics in personalized phantoms of healthy and stenosed coronary arteries. The proposed set-up was fine-tuned with the aim of obtaining a compact, flexible, low-cost test-bench for biomedical applications. Technically, velocity vector fields were measured adopting a so-called smart-PIV approach, consisting of a smartphone camera and a low-power continuous laser (30 mW). Experiments were conducted in realistic healthy and stenosed 3D-printed phantoms of left anterior descending coronary artery reconstructed from angiographic images. Time resolved image acquisition was made possible by the combination of the image acquisition frame rate of last generation commercial smartphones and the flow regimes characterizing coronary hemodynamics (velocities in the order of 10 cm/s). Different flow regimes (Reynolds numbers ranging from 20 to 200) were analyzed. The smart-PIV approach was able to provide both qualitative flow visualizations and quantitative results. A comparison between smart-PIV and conventional PIV (i.e., the gold-standard experimental technique for bioflows characterization) measurements showed a good agreement in the measured velocity vector fields for both the healthy and the stenosed coronary phantoms. Displacement errors and uncertainties, estimated by applying the particle disparity method, confirmed the soundness of the proposed smart-PIV approach, as their values fell within the same range for both smart and conventional PIV measured data (≈5% for the normalized estimated displacement error and below 1.2 pixels for displacement uncertainty). In conclusion, smart-PIV represents an easy-to-implement, low-cost methodology for obtaining an adequately robust experimental characterization of cardiovascular flows. The proposed approach, to be intended as a proof of concept, candidates to become an easy-to-handle test bench suitable for use also outside of research labs, e.g., for educational or industrial purposes, or as first-line investigation to direct and guide subsequent conventional PIV measurements.

## 1 Introduction

In the last two decades, particle image velocimetry (PIV) has become a standard technique for the reliable *in vitro* quantitative characterization of fluid dynamics in implantable devices such as prosthetic heart valves (Manning et al., 2003; Leo et al., 2006; Dasi et al., 2007; Kaminsky et al., 2007; Dasi et al., 2008; Ge et al., 2008; Hasler et al., 2016; Hasler and Obrist, 2018; Becsek et al., 2020) and stents (Charonko et al., 2009; Charonko et al., 2010; Raben et al., 2015; Brindise et al., 2017; Freidoonimehr et al., 2021a), in blood recirculating devices such as extracorporeal membrane oxygenators, mechanical circulatory supports, blood pumps and hemodialysis systems (Giridharan et al., 2011; Raben et al., 2016; Malinauskas et al., 2017), as well as in idealized and realistic phantoms of healthy and diseased vessels (Bluestein et al., 1997; Brunette et al., 2008; Ford et al., 2008; Kefayati and Poepping, 2013; Büsen et al., 2017; Shintani et al., 2018; DiCarlo et al., 2019; Salman et al., 2019; Medero et al., 2020; Freidoonimehr et al., 2021b).

Despite the proven capability of conventional PIV test benches in characterizing internal flows, their adoption in both research and industrial laboratories is hampered by the cost of the components (rough order of magnitude estimate of 100 k€). In recent years, attempts have been made to propose alternative PIV solutions based on low-cost components, thus overcoming cost-related barriers (Cierpka et al., 2016; Aguirre-Pablo et al., 2017; Käufer et al., 2021; Minichiello et al., 2021). In this respect, the imaging system embedded in smartphones have captured the attention of researchers as potential substitute of high-speed cameras adopted in conventional PIV, leveraging the latest smartphone technological advancements and their relatively low cost. The first generalized attempt of a smartphone-based PIV approach defined the set-up requirements in terms of acquisition frequency and optical magnification as a function of the flow velocity, limiting the applicability to low velocity flows or coarse spatial resolutions (Cierpka et al., 2016). Subsequently, the use of smartphone-based PIV systems was extended to 3D measurements, synchronizing the acquisition from four smartphones in tomo-PIV configuration (Aguirre-Pablo et al., 2017).

Further drawbacks affecting conventional PIV systems are related to the use of a high-power (double-) pulsed laser to illuminate the volume of interest, usually characterized by high costs, high energy consumption and burdensome maintenance. Moreover, such lasers require complex and expensive set-ups to guarantee synchronization (Chételat and Kim, 2002; Willert et al., 2010, among others) and safety requirements (EN 207 in EU; ANSI z136 in US). Regarding the latter, among the most commonly adopted energy sources for flow fields illumination in conventional PIV systems are 200 mJ pulsed lasers, belonging to Class 4, which is the most hazardous class of laser according to the international standard IEC 60825-1. These limiting factors motivated the adoption of less expensive and less hazardous low-energy light sources, such as high-performance LEDs (Willert et al., 2010; Aguirre-Pablo et al., 2017) or continuous wave (*cw*) lasers (Cierpka et al., 2016).

Taken together, these considerations underline the theoretical benefits offered by a PIV system relying on the combined use of smartphone cameras and low-energy light sources in terms of costs, simplicity, and safety. In the followings, we will refer to such a PIV system as “smart-PIV”, as introduced in a recent study presenting a smartphone-based PIV dedicated software application (Cierpka et al., 2021). To date, the practical feasibility, range of applicability and related performances of a smart-PIV approach in biomedical applications have not yet been clearly defined. Whilst Cierpka et al., 2016 demonstrated the feasibility of PIV measurements of planar velocity vector fields generated by a free water jet using a smartphone camera with acquisition rate of 240 Hz and a *cw* laser in absence of synchronization systems, the adoption of a smart-PIV approach for cardiovascular flows-related applications is still unexplored. The rapid development of modern smartphone cameras has recently led to an increase in the frame rate up to 1.920 Hz, thus enabling their use for velocities in the order of 80–100 cm/s (Nichols et al., 2011). Accordingly, the objective of the present work was to demonstrate the feasibility of a smart-PIV approach to the characterization of arterial flows in realistic physical models. To this aim, a last generation commercial smartphone was used as image acquisition device in combination with a low-power *cw* laser to measure the velocity vector field in realistic phantoms of healthy and stenosed coronary arteries at various flow regimes. The fluid dynamics characterization obtained adopting the smart-PIV system was then compared with the results obtained by adopting conventional PIV, which is considered the gold-standard experimental technique for bioflows characterization. The study was completed by the analysis of the uncertainty affecting the measured flow fields.

## 2 Materials and methods

### 2.1 PIV measurements of coronary flows: Reference framework

A survey of the literature on PIV characterizations of coronary flows was preliminarily conducted to delineate the reference framework for the operating conditions to be set in the present study. The results of the survey on conventional PIV measurements in coronary artery phantoms are summarized in Table 1, where basic information on flow regimes, adopted PIV settings, and indication whether the studies were carried out relying on a scale factor according to the Buckingham theorem of fluid dynamics similitude (Buckingham, 1914) are detailed. From Table 1, it emerges that previous investigations considered coronary flow regimes characterized by Reynolds numbers at the inflow section of the coronary phantoms $Re_{inflow}$ < 450, which in turn correspond to average velocity magnitude values on the order of magnitude of tens of cm/s. Moreover, PIV measurements were based on conventional dual-frame acquisitions, performed setting time intervals ( ∆t ) of order of magnitude in a range from tens to thousands µs, and image resolutions (*l*_*o*_) from 1 to 50 µm/pixel.

**TABLE 1:** Flow parameters and PIV spatial and temporal resolutions adopted in *in vitro* PIV experiments on coronary flows. $d_{a}$ , inlet artery model diameter; $\left|V\right|$ , mean inflow velocity $Re_{inflow}$ , inflow Reynolds number; ∆t , time interval between two consecutive frames; $l_{o}$ , image resolution; M , magnification factor.

	Scale factor	$d_{a}$ [mm]	$\left|V\right|$ [cm/s]	${Re}_{inflow}$	∆t [μs]	$l_{o}$ [μm/pixel]	*M*
Raz et al. (2007)	5	15	4.4*	200	33367	37.50*	0.3*
Brunette et al. (2008)	6.35	19.1	13.0	194	200	47.00	—
Charonko et al. (2010)	1	3.0; 4.0	14.9*–37.2*	160; 300	25–70	1.73; 2.31	4.0*; 3.0*
Kabinejadian et al. (2014)	1	4.0	9.3*	79	—	7.50*	—
Raben et al. (2015)	1	4.0	11.5*	120*	200	4.78; 7.33	1.5*; 1.0*
Brindise et al. (2017)	1	4.0	14.3*–42.8*	150–450	200	7.04; 7.73	1.7*; 1.6*
Freidoonimehr et al. (2021a)	2	6.4	10.9*	210	70	2.50; 4.32	2.6*; 1.5*
Freidoonimehr et al. (2021b)	2	6.1	6.6*	120*	66667	4.52	1.5*
Current experiment (smart-PIV)	1	3.0	5.0–50.0	20–200	960	28.00	0.05

*value derived from the reported data.

### 2.2 Basic principles of conventional PIV and smart-PIV

According to the principle of PIV, the measured velocity is determined by the ratio of the ensemble particle displacement in the object plane in physical space, $∆x_{o}$ , and the time interval ∆t occurring between the acquisition of two consecutive frames. From a practical viewpoint, being $∆x_{i\;}$ the image displacement projected to the image acquisition camera sensor, the relationship between $∆x_{o}$ and $∆x_{i\;}$ can be easily obtained through the knowledge of the magnification factor *M* of the adopted optical system. The quantity $∆x_{i}$ is frequently expressed in pixels as $∆x_{i,px}$ , so that, knowing the pixel size dimension $l_{i}$ (µm/pixel) for the adopted camera sensor, the measured velocity can be expressed as:

$$V=\frac{∆x_{o\;}}{∆t}=\frac{∆x_{i\;}}{M∆t}=\frac{∆x_{i,px\;}l_{i}}{M∆t}.$$ (1)

A value of 10–15 pixel has been recommended in the literature for the image particle displacement (Raffel et al., 2018). Therefore, in the dual-frame modality at the basis of conventional PIV, $∆x_{i,px}$ can be adjusted to optimize the velocity measurement by setting appropriate values for the time interval ∆t and for the magnification factor *M*.

**FIGURE 1:**
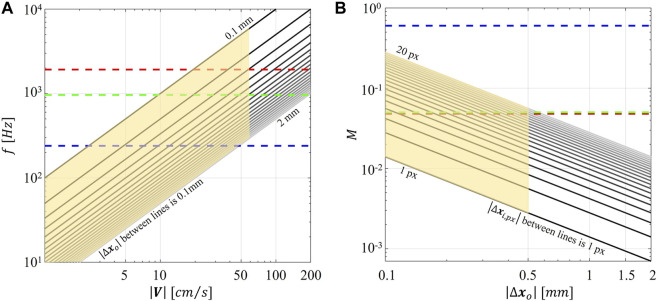
**(A)** Magnitude of particle displacement in the object plane ( $\left|∆x_{o}\right|$ ) depending on the magnitude of flow velocity ( $\left|V\right|$ ) and the acquisition frequency ( f ), assuming pixel sizes of 1.4 μm, typical of current smartphone cameras. **(B)** Magnitude of particle displacement in the image plane ( $\left|∆x_{i,px}\right|$ ) depending on the magnitude of the particle displacement in the object plane ( $\left|∆x_{o}\right|$ ) and the magnification factor (*M*), assuming a pixel size of 1.4 μm. Dashed lines correspond to the Huawei Mate Pro 30 (red dotted line), the Samsung Galaxy S9+ (green dotted line), used in the present work, and the iPhone 13 Pro (blue dotted line). The highlighted area represents the measurement feasibility range in the smart-PIV configuration.

Unlike conventional PIV, the smart-PIV system is based on a continuous single-frame modality for image acquisition similar to the one adopted for high-speed camera acquisitions (Hain and Kähler, 2007). In single-frame modality, particle displacement in the physical space is given by $∆x_{o}=V/f$ , where f is the image acquisition frame rate. According to the approach proposed by Cierpka et al., 2016 and assuming a pixel size of 1.4 μm (typically characterizing the last generation of smartphone cameras), diagrams can be drawn to relate the flow velocity magnitude of interest for coronary flows $\left|V\right|$ , the displacement that can be measured in the flow field $∆x_{o}$ , and the smartphone camera acquisition frame rate f (Figure 1A). Diagrams in Figure 1 were built considering three different image acquisition frame rates f values, corresponding to the following three smartphones currently available on the market: Huawei Mate Pro 30 ( f = 1920 Hz), Samsung Galaxy S9+ (the smartphone adopted in the present study, f = 960 Hz), and iPhone 13 Pro ( f = 240 Hz, as for the iPhone 6 adopted in a previous study (Cierpka et al., 2016)). From Figure 1, it clearly emerges that only smartphones with f equal or greater than 960 Hz can be used effectively to measure velocities up to 200 cm/s. Figure 1B also reports the measurable image displacement in pixels $∆x_{i,px}$ as a function of the displacement in physical space $∆x_{o}$ and of the magnification factor *M* of the adopted smartphone optical system. The only way to increase image magnification is by reducing the distance between the smartphone camera and the measurement plane, until the image goes out of focus. Therefore, the maximum magnification is an inherent characteristic of the camera and defines the upper limit of the validity region of the diagrams. Considering an image acquisition rate of 960 Hz, flow velocities in the range 10–50 cm/s correspond to fluid displacements within the range 0.1–0.5 mm (Figure 1), which in turn corresponds, with a *M* value set to 0.05, to image displacement values $∆x_{i,px}$ in the range 4–16 pixels. This demonstrates that PIV measurements using smartphone cameras can be performed on the spectrum of fluid velocities characterizing coronary flows.

### 2.3 Smart-PIV and conventional PIV set-ups

**FIGURE 2:**
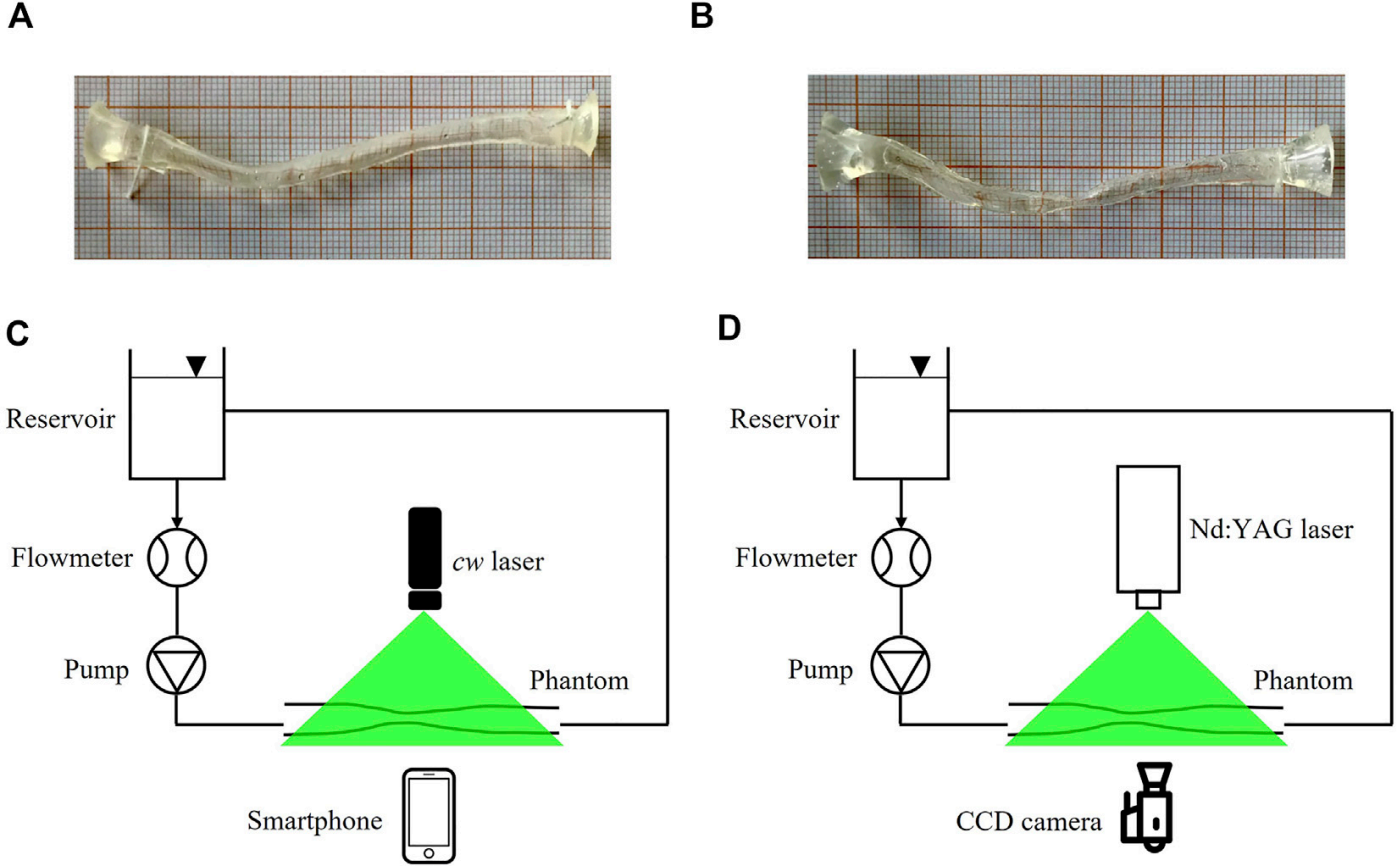
**(A**,**B)** pictures of the healthy LAD **(A)** and stenosed LAD **(B)** phantoms, scale 1:1. **(C**,**D)** experimental set-up for smart-PIV **(C)** and conventional PIV **(D)** experiments. *cw*: continuous wave; CCD: charge-coupled device.

PIV measurements were performed in two flexible silicone phantoms manufactured by Elastrat (Geneva, Switzerland). The first phantom represented a patient specific replica of a 3 mm healthy left anterior descending (LAD) coronary artery (Figure 2A), reconstructed from angiographic images, as detailed elsewhere (Lodi Rizzini et al., 2020). The second phantom was obtained starting from the healthy LAD geometry, where a 67% diameter stenosis was artificially generated by imposing a local reshaping of the lumen geometry through the open-source tool morphMan (Kjeldsberg et al., 2019) (Figure 2B). The two phantoms were in scale 1:1. The refractive index of the adopted material was equal to 1.43.

The sketch of the two experimental set-up configurations used for flow visualizations and PIV measurements is presented in Figures 2C, D. The two configurations adopted the same hydraulic circuit, where 500 ml of working fluid was handled by a DC current pump (RS Components, Corby, United Kingdom) with a nominal power of 1.62 W controlled by a power supply to assure a constant flow rate. One reservoir at atmospheric pressure decoupled the upstream pumping system from the phantom. Flow rate measurements were obtained by an in-line ultrasound flowmeter (Transonic, Ithaca, United States) characterized by an accuracy of ±10% (Figures 2C, D). The adopted working fluid was a glycerol-water (40:60 in volume) solution with a dynamic viscosity of 3.7 cP (Segur and Oberstar, 1951). Polyamide poly (methyl methacrylate) particles (density 1030 kg/m^3^, diameter 60 µm) were used.

PIV measurements were carried out in steady-state conditions at different flow regimes in both healthy and stenosed LAD phantoms (Doucette et al., 1992; Kessler et al., 1998; Johnson et al., 2008). The investigated flow regimes are summarized in Table 2, where the Reynolds numbers evaluated at the inflow section of the phantoms ( $Re_{inflow}$ ) are reported together with the corresponding flow rate values ( Q ). For the stenosed LAD model, the Reynolds numbers at the stenosis ( $Re_{stenosis}$ ) are also reported.

**TABLE 2:** Inlet flow rate ( Q ) and Reynolds numbers characterizing the investigated flow regimes at the inlet section ( $Re_{inflow}$ ) and at the stenosis ( $Re_{stenosis}$ ) for the stenosed LAD phantom. The time intervals ( ∆t ) between consecutive frames adopted in the conventional PIV approach are also presented.

${Re}_{inflow}$	${Re}_{stenosis}$	Q [ml/min]	∆t [μs] conventional PIV
Healthy LAD			
43	—	20	1993
85	—	40	997
171	—	80	498
213	—	100	400
Stenosed LAD			
21	64	10	900
64	192	30	300
107	320	50	180
171	512	80	112

In the smart-PIV set-up, the smartphone Samsung Galaxy S9+ was adopted as image capture system because of its acquisition frame rate ( f = 960 Hz, $f_{\#}$ = 2.4, being $f_{\#}$ the used f-stop of the objective) in the so called “super-slow-motion” modality. A low-power (30 mW, λ = 532 nm) *cw* laser was adopted as light source for illuminating the fluid domain of interest in the smart-PIV set-up. The thickness of the light sheet was approximately 1 mm. Due to limited storage capability of the smartphone 20 consecutive acquisitions were recorded per each one of the investigated flow regimes (Table 2). Each one of the 20 acquisitions consisted of 180 consecutive frames.

In the conventional PIV set-up, the image capture system was composed by a HiSense Zyla camera (CMOS, 2560 × 2160 pixels) with a macro-objective Zeiss Milvus 50 mm ( $f_{\#}$ = 16). The light source for the illumination of the flow field of interest was composed by a dual pulsed Nd:YAG laser (200 mJ, 15 Hz, λ = 532 nm) and a synchronization unit. The thickness of the light sheet was set to ≈1 mm to minimize the out-of-plane motion. As listed in Table 2, for each one of the investigated flow regimes image pairs were acquired setting the time intervals ∆t to obtain particle image displacements below 10 pixels, according to previous studies (Raffel et al., 2018). The statistical convergence was assured acquiring 1000 image pairs per each investigated flow regime.

For comparison purposes, the imaging parameters in smart-PIV and conventional PIV measurements were selected to guarantee the same investigated field of view (Table 3).

**TABLE 3:** Imaging parameters of smart and conventional PIV: resolution, magnification factor ( M ), pixel size in the image plane ( $l_{i}$ ), pixel size in the object plane ( $l_{o}$ ), diffraction limited image diameter ( $d_{diff}$ ).

	Resolution [pixel]	*M*	$l_{i}$ [μm/pixel]	$l_{o}$ [μm/pixel]	$d_{diff}$ [μm]
Smart-PIV	1280 × 720	0.05	1.4	28.0	3.3
Conventional PIV	2560 × 2160	0.5	6.5	13.0	31.1

### 2.4 Image processing

Smart-PIV and conventional PIV acquired raw images were preliminarily pre-processed in MATLAB environment (MathWorks, Natick, MA, United States) to remove background noise by subtracting the mean intensity value of the PIV image sequence. For flow visualization purposes, seeding particle trajectories were reconstructed over 180 consecutive frames acquired with the smart-PIV approach. Since seeding particle motion between two consecutive acquired frames is sufficiently small, the reconstruction of particle trajectories can be done by calculating the root mean square intensity values of the pre-processed images along the frame series. This method allowed to obtain similar results to those given by the common technique based on long exposure imaging (Merzkirch, 2012).

The velocity vector fields were extracted applying the ensemble correlation method to the pre-processed images, a method indicated for analyzing sparsely seeded steady flows (Santiago et al., 1998; Meinhart et al., 2000). Technically, the ensemble correlation is based on the analysis of a series of sparsely seeded images and on the calculation of their correlation matrices. These matrices are then averaged to give a high-resolution velocity vector field characterized by a signal-to-noise ratio which can be obtained by the standard cross-correlation only through a coarser resolution (Meinhart et al., 2000). The ensemble correlation was performed adopting the toolbox PIVlab (Thielicke and Stamhuis, 2014). Interrogation windows (IWs) of 16 and 24 pixels were considered on smart-PIV and on conventional PIV acquired images, respectively, thus obtaining IWs in the object plane of approximately the same dimension (0.50 and 0.51 mm, respectively). A 50% window overlapping was applied.

### 2.5 Error and uncertainty estimation

PIV measurements are affected by uncertainty. The sources of such an uncertainty can be identified in 1) the components of the acquisition system, 2) the peculiar features of the analyzed flow field (e.g., high velocity gradients and out-of-plane motion), and 3) the image processing strategy. For a comprehensive discussion, the reader is referred to Raffel et al., 2018 and Sciacchitano, 2019, among others. Since the field of view and adopted processing strategy were the same in smart-PIV and in conventional PIV approaches, the analysis performed in this study is intended to highlight the uncertainty associated with those smart-PIV components that are not part of the conventional PIV system.

In this study, the particle disparity method (Sciacchitano et al., 2013) was employed to evaluate and compare the budget of estimated error and uncertainty affecting smart-PIV and conventional PIV measurements in the LAD phantoms. Technically, particle disparity is an *a posteriori* method that quantifies from images the uncertainty affecting particles displacement, i.e. the major contributor to the velocity uncertainty (Sciacchitano, 2019). In detail, the budget of uncertainty associated with PIV velocity measurements can be estimated adopting a Taylor series expansion for evaluating the single contributions to uncertainty given by the quantities expressing fluid velocity (Sciacchitano, 2019). As detailed in Section 2.2, these are the ensemble displacement of a group of tracing particles measured in the image plane ∆x , the magnification factor *M*, and the time interval ∆t . As reported elsewhere, uncertainty related to the magnification factor *M* can be considered negligible when calibration is properly conducted (Sciacchitano, 2019). Uncertainty related to ∆t is of the order of 1 ns for Nd:YAG lasers (Lazar et al., 2010; Bardet et al., 2013), thus negligible when compared to the ∆t values adopted in this study (which are of the order of 100 μ s). Consequently, the major contribution to the budget of uncertainty affecting velocity is given by the uncertainty affecting particle displacement.

**FIGURE 3:**
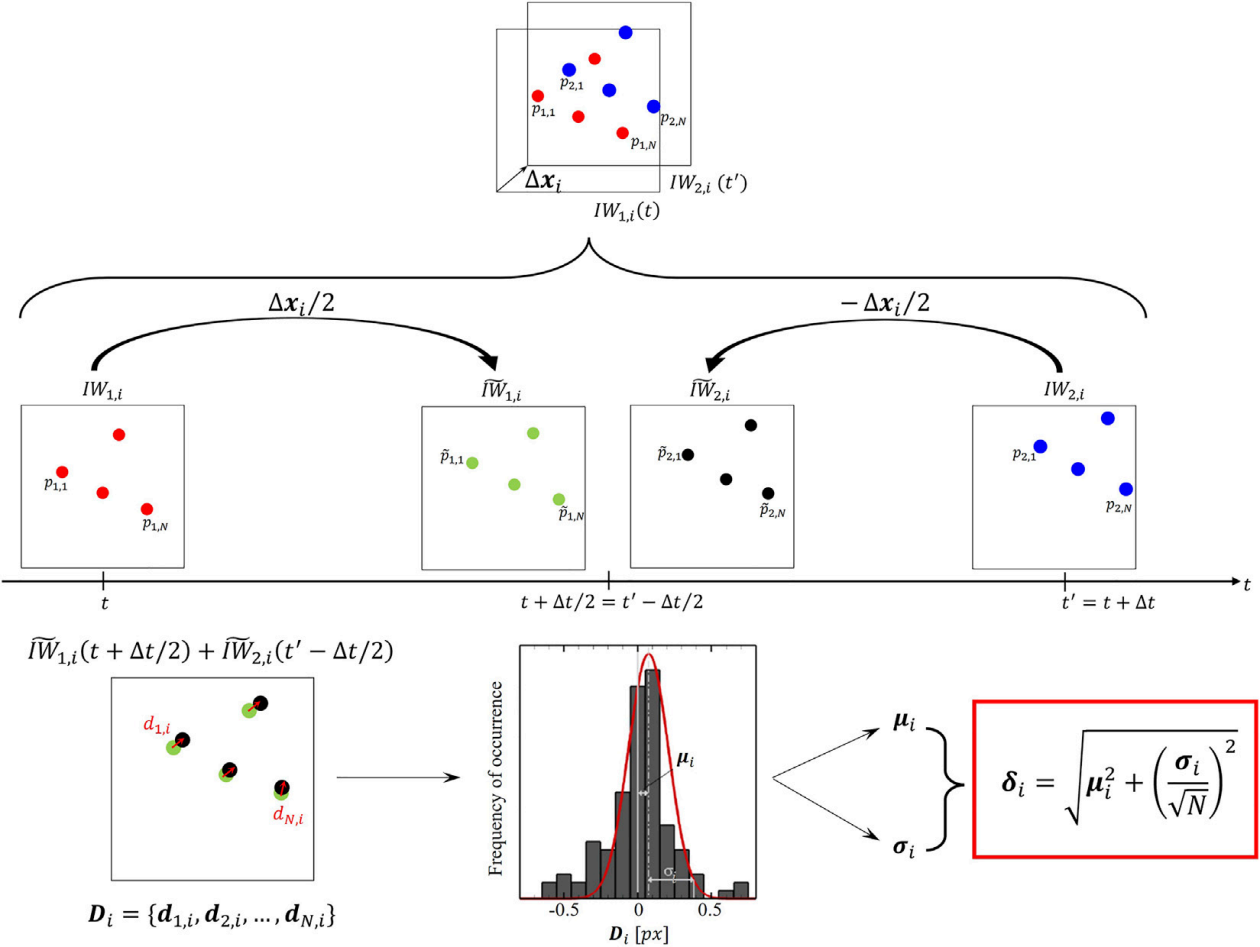
Schematic of the particle disparity method applied to the *i*-th couple of interrogation window $IW_{1,i}$ and $IW_{2,i}$ on images $I_{1}\left(t\right)$ and $I_{2}\left(t^{\prime }\right)$ , respectively. The particle ensemble displacement in $∆t=t^{\prime }-t$ within the *i*-th couple of interrogation windows as measured by PIV is $∆x_{i}$ . ${\tilde{IW}}_{1,i}\left(t+∆t/2\right)$ and ${\tilde{IW}}_{2,i}\left(t^{\prime }-∆t/2\right)$ are the reconstructed image windows used to evaluate the disparity set ( $D_{i}$ ). The error on the displacement vector on the i-th interrogation window ( $\delta_{i}$ ) is evaluated computing the mean ( $\mu_{i}$ ) and the standard deviation ( $\sigma_{i}$ ) of the components of $D_{i}$ .

Let us consider two consecutive frames $I_{1}\left(t\right)$ and $I_{2}\left(t^{\prime }\right)$ , separated by a time interval ∆t ( $t^{\prime }=t+∆t$ ), and a displacement field ∆x obtained from PIV analysis. $I_{1}$ and $I_{2}$ are divided into the same number of interrogation windows ${IW}_{1,i}$ and ${IW}_{2,i}$ ( i=1,…,F with F total number of interrogation windows), respectively. From $I_{1}\left(t\right)$ and $I_{2}\left(t^{\prime }\right)$ the displacements field ∆x can be evaluated according to the ensemble correlation method, for the case under study. Then, all seeding particles in ${IW}_{1,i}$ and ${IW}_{2,i}$ are shifted by $+∆x_{i}/2$ , and $-∆x_{i}/2$ , respectively, where $∆x_{i}$ is the i *-th* ensemble displacement vector measured by PIV on the couple ${IW}_{1,i}$ and ${IW}_{2,i}$ the result is the reconstruction of displacement of single particles at intermediate time ∆t/2 between t and $t^{\prime }$ (as depicted in ${\tilde{IW}}_{1,i}\left(t+∆t/2\right)$ and ${\tilde{IW}}_{2,i}\left(t^{\prime }-∆t/2\right)$ frames in Figure 3). Ideally PIV measurements lead to a perfect overlapping of the corresponding image particles in ${\tilde{IW}}_{1,i}\left(t+∆t/2\right)$ and ${\tilde{IW}}_{2,i}\left(t^{\prime }-∆t/2\right)$ . However, since the PIV velocity field is only an approximation of the particle motion, image particles in the two reconstructed windows ${IW}_{1,i}$ and ${IW}_{2,i}$ will not exactly overlap. The residual distance between each matched seeding particle k can be obtained, with k=1,…,N being N number of particles within the interrogation window couple. Such residual distance, the so-called disparity vector $d_{k}$ , will contribute as the k *-th* component of the disparity set $D_{i}$ characterizing the couple of interrogation windows ${IW}_{1,i}$ and ${IW}_{2,i}$ . The mean value $\mu_{i}$ and the standard deviation $\sigma_{i}$ of the components of the disparity set $D_{i}$ measure the systematic and the precision error of the PIV measurement within the i *-th* couple of interrogation windows, respectively, ultimately defining the error estimation $\delta_{i}$ of the measured seeding particles displacement, according to Sciacchitano et al., 2013:

$$\delta_{i}=\sqrt{\mu_{i}^{2}+{\left(\frac{\sigma_{i}}{\sqrt{N}}\right)}^{2}}$$ (2)

After repeating the above-described procedure over the *F* interrogation windows, an estimated error field with the same dimensions of the input velocity field can be obtained. Furthermore, here the displacement uncertainty $U_{∆x_{i}}$ was determined according to (Sciacchitano et al., 2013):

$$U_{∆x_{i}}=k\;\delta_{i}$$ (3)

where *k* is a coverage factor whose value is around 2.1, to achieve 95% confidence level for small *N* values as occurring in PIV interrogation boxes (Coleman and Steele, 2009).

## 3 Results

### 3.1 Flow visualizations

**FIGURE 4:**
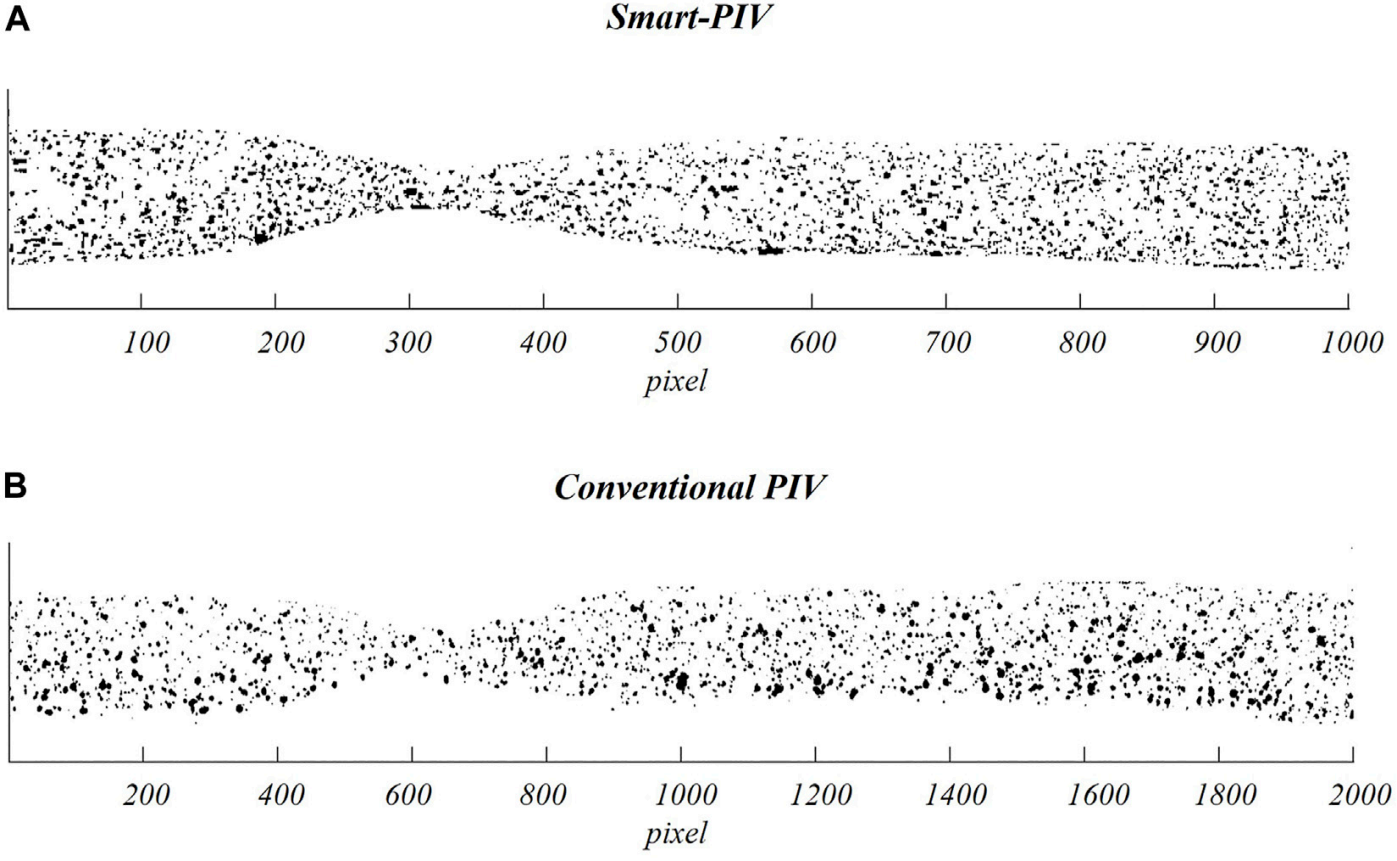
Examples of pre-processed images (i.e., after subtraction of the mean intensity value of the PIV image sequence) acquired on the stenosed LAD phantom adopting smart-PIV **(A)** and conventional PIV **(B)** systems.

Two examples of pre-processed images acquired with smart-PIV and conventional PIV are displayed in Figure 4.

**FIGURE 5:**
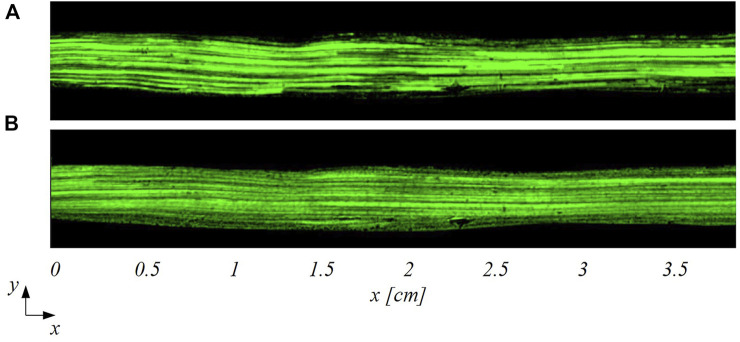
Particle trajectories-based visualization of the flow patterns in the healthy LAD at two different inflow Reynolds numbers: **(A)** $Re_{inflow}$ = 43; **(B)** $Re_{inflow}$ = 213.

**FIGURE 6:**
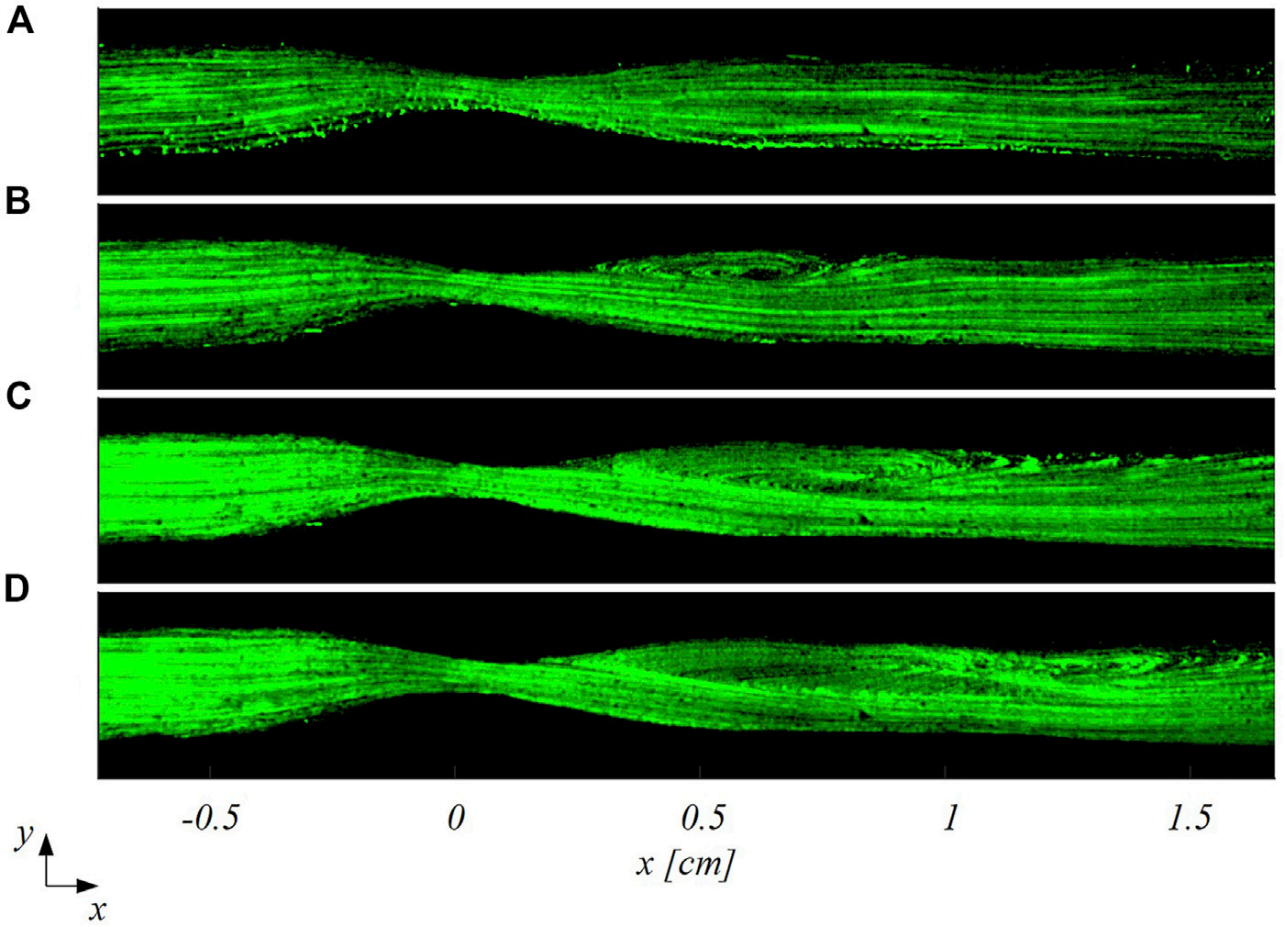
Particle trajectories-based visualization of the flow patterns in the stenosed LAD at four different inflow Reynolds numbers: **(A)** $Re_{inflow}$ = 21; **(B)** $Re_{inflow}$ = 64; **(C)** $Re_{inflow}$ = 107; **(D)** $Re_{inflow}$ = 171.

The smart-PIV images were processed for flow visualization purposes. The reconstructed trajectories of seeding particles unveil the main flow features within the healthy and stenosed LAD phantoms. In detail, particle trajectories evolved unperturbed in the healthy LAD phantom at all investigated flow regimes, as expected (Figure 5). For this reason, Figure 5 depicts only the cases at $Re_{inflow}$ = 43 and $Re_{inflow}$ = 213. In the stenosed LAD phantom, particle trajectories visualization highlights the presence of a flow recirculation region downstream of the stenosis which becomes larger as $Re_{inflow}$ increases (Figure 6). More in detail, at $Re_{inflow}$ = 21 no flow separation occurs (as expected, Figure 6A), while at increasing Reynolds number typical flow features of stenosed coronary hemodynamics emerge from particle traces visualization (Figures 6B–D): 1) a high-velocity jet-like flow configuration at the stenosis; 2) a recirculation region whose extension increases longitudinally with greater Reynolds number, in agreement to previous *in vitro* experiments (Geoghegan et al., 2013; Brunette et al., 2008, among others). Moreover, flow visualizations in Figures 6B–D clearly depict the interface between flow jet and the recirculation regions, with the former becoming thinner at increasing Reynolds numbers. In addition, at $Re_{inflow}$ = 64, a well-defined reattachment point can be observed (Figure 6B), which is located more downstream when increasing the $Re_{inflow}$ to 107 (Figure 6C). At $Re_{inflow}$ = 171, the flow field is completely separated (Figure 6D). These visualizations highlight that the smart-PIV system is able to capture the expected flow features in the LAD phantoms.

### 3.2 Comparison between smart-PIV and conventional PIV velocity measurements

**FIGURE 7:**
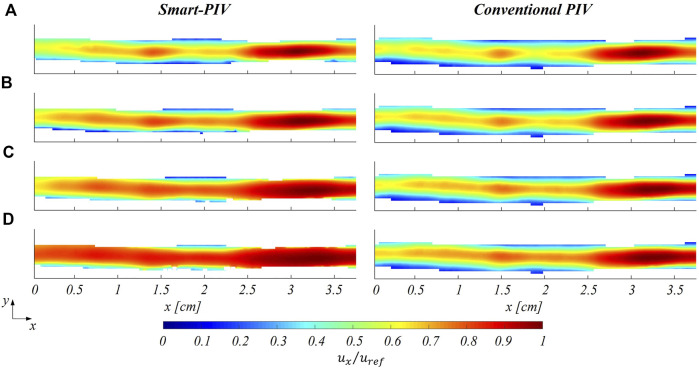
Normalized mean streamwise velocity contours for smart (left panel) and conventional (right panel) PIV at four different flow regimes: **(A)** $Re_{inflow}$ = 43, **(B)** $Re_{inflow}$ = 85, **(C)** $Re_{inflow}$ = 171, **(D)** $Re_{inflow}$ = 213 for the healthy LAD phantom. The mean stream-wise velocity ( $u_{x}$ ) is normalized to the maximum streamwise velocity ( $u_{ref}$ ).

**FIGURE 8:**
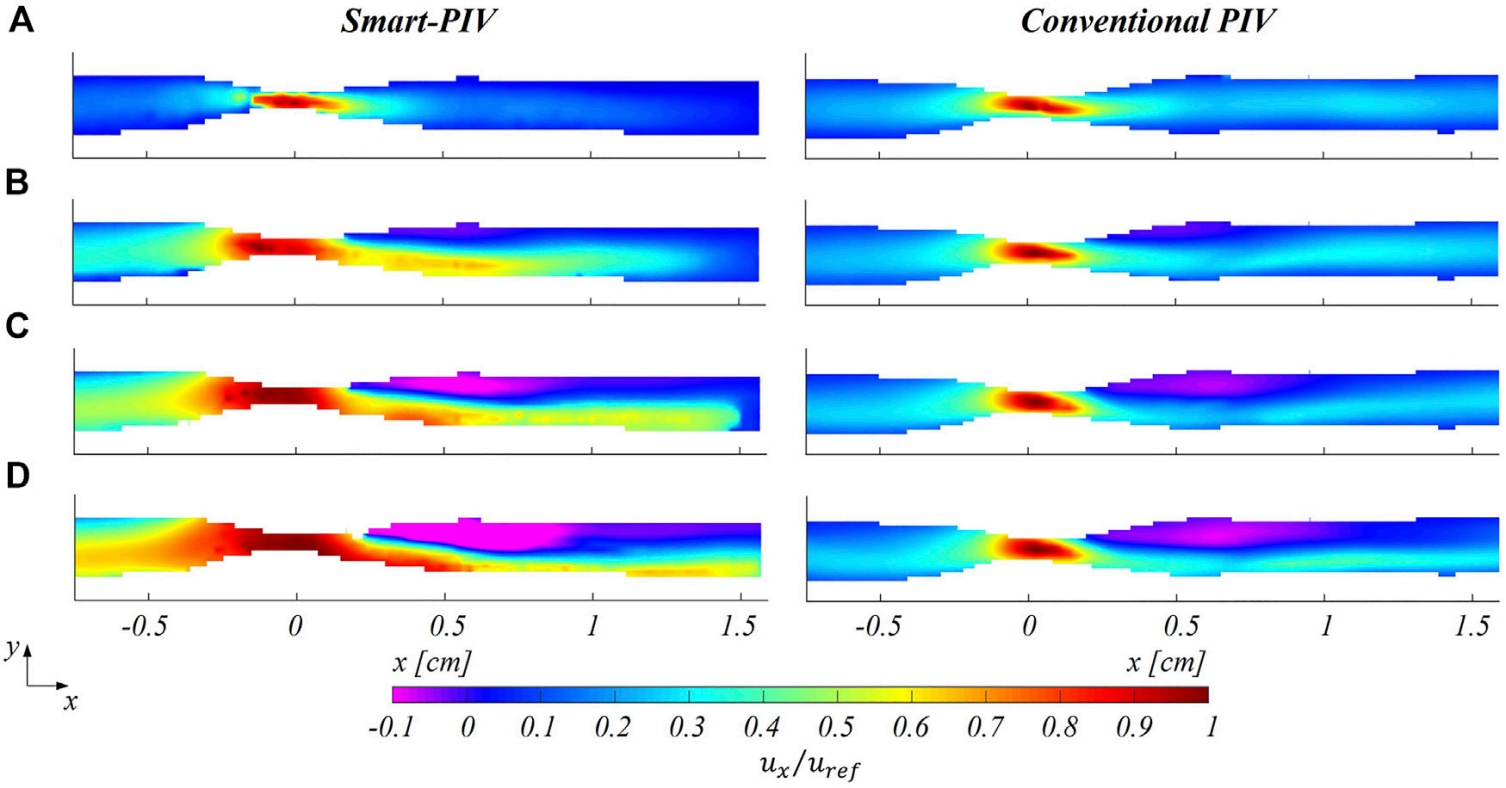
Normalized mean streamwise velocity contours for smart (left panel) and conventional (right panel) PIV at four different flow regimes: **(A)** $Re_{inflow}$ = 21; **(B)** $Re_{inflow}$ = 64; **(C)** $Re_{inflow}$ = 107; **(D)** $Re_{inflow}$ = 171 for the stenosed LAD phantom. The mean stream-wise velocity ( $u_{x}$ ) is normalized to the maximum velocity ( $u_{ref}$ ).

In all models, the streamwise velocity component ( $u_{x}$ ) is predominant over the spanwise velocity component ( $u_{y}$ ) in most of the fluid domain (Supplementary Figures S1, S2). Thus, the performance of the smart-PIV system was compared to conventional PIV in terms of axial velocity $u_{x}$ normalized with respect to the maximum streamwise velocity $\left(u_{ref}\right)$ . In the healthy LAD phantom, smart-PIV and conventional PIV measurements highlighted similar flow features at the four investigated flow regimes (Figure 7), the former being able to replicate the performance of the conventional PIV measurement technique. In the stenosed LAD phantom, smart-PIV and conventional PIV measurements were in substantial agreement in detecting the flow separation phenomena occurring downstream of the stenosis (Figure 8). Overall, the smart-PIV approach succeeded in capturing the fluid structures identified by the conventional PIV. A detailed quantitative analysis on streamwise velocity profiles is provided in the Supplementary Material (Supplementary Figures S3–S6).

As expected, an underestimation of the highest velocity values affected smart-PIV measurements, when compared to conventional PIV: in the healthy phantom and in the proximal segment of the stenosed phantom, such an underestimation was bounded below the 20% and increased with the $Re_{inflow}$ value. In the throat of the stenosis and the region immediately downstream, the smart-PIV velocity underestimation increased up to 40% at the highest Reynolds number investigated (Figure 8D, $Re_{inflow}$ = 171), where high velocity gradients characterized the flow field in the stenosis throat. The underestimation in smart-PIV velocity data can be ascribed to the image particle blurring affecting smart-PIV images but not conventional PIV images (Supplementary Figure S7). Particle blurring resulted from the combination of the high flow velocity ( $u_{x}$ > 40–45 cm/s), the continuous light source and the acquisition frame rate of the smartphone camera ( f = 960 Hz), at the adopted magnification factor (*M* = 0.05). The comparison of the normalized spanwise velocity contours for both healthy and stenosed LAD phantoms is reported in the Supplementary Figures S8, S9.

### 3.3 Error and uncertainty estimation

**FIGURE 9:**
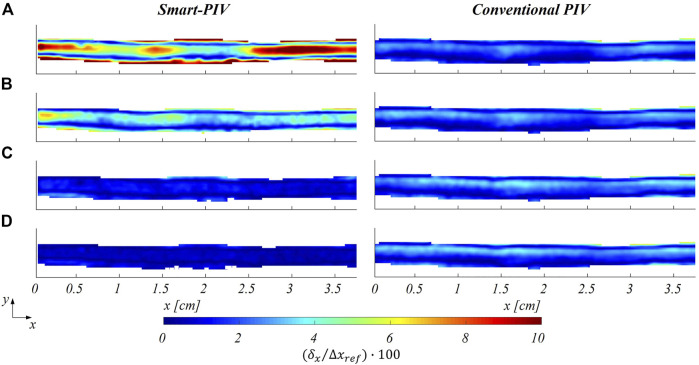
Color maps of smart (left panel) and conventional (right panel) PIV displacement errors along the streamwise flow direction $\delta_{x}$ , normalized to the maximum streamwise particle displacement ( $∆x_{ref}$ ) at four different flow regimes: **(A)** $Re_{inflow}$ = 43, **(B)** $Re_{inflow}$ = 85, **(C)** $Re_{inflow}$ = 171, **(D)** $Re_{inflow}$ = 213 for the healthy LAD phantom.

**FIGURE 10:**
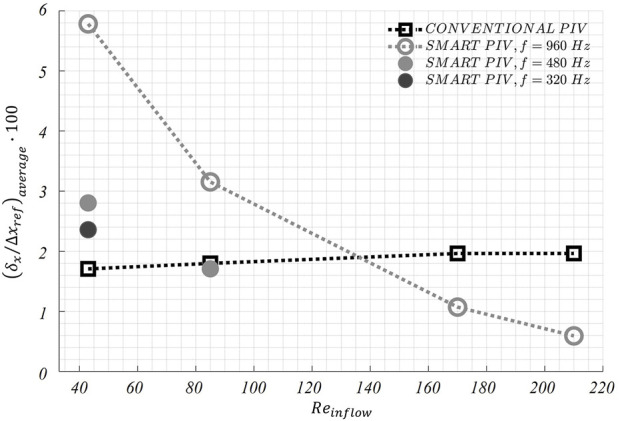
Variations of the average displacement error along the streamwise direction $\left(\delta_{x}\right.$ ) normalized by maximum streamwise particle displacement ( $∆x_{ref}$ ) as a function of the inflow Reynolds number ( $Re_{inflow}$ ) in the healthy LAD phantom. For smart PIV, the normalized average displacement error $\delta_{x}/∆x_{ref}$ is evaluated also by virtually reducing the image acquisition frequency to 480 Hz (for $Re_{inflow}$ = 43, $Re_{inflow}$ = 85) and 320 Hz (for $Re_{inflow}$ = 43).

The distribution of the estimated displacement errors along the streamwise direction $\delta_{x}$ , normalized to the maximum streamwise particle displacement ${∆x}_{ref}$ , is presented in Figure 9 for the healthy LAD phantom. It can be noticed that conventional PIV measurements were affected by normalized estimated displacement errors lower than 5%. Moreover, these errors exhibited a moderate dependence on the investigated flow regimes by virtue of the tuning of the dual frame acquisition time interval ∆t . On the opposite, smart-PIV measurements presented decreasing normalized estimated displacement error values with increasing $Re_{inflow}$ values. As shown in Figure 9, the normalized estimated displacement errors along the streamwise flow direction $\delta_{x}/{∆x}_{ref\;}$ values were larger than 5% for $Re_{inflow}$ < 85, while they decreased below 2% at higher *Re*_*inflow*_. The dependence of smart-PIV normalized estimated displacement errors on flow regime can be ascribed to the fixed acquisition frame rate of the smartphone camera, which does not allow to tune the dual frames time interval at slower flow regimes. However, it is possible to obtain larger particle displacements ∆x by keeping one every two or three acquired frames. In this way, the image acquisition frame rate of the smartphone camera is virtually reduced from 960 Hz to 480 Hz and 320 Hz, respectively. This operation reduced the normalized estimated displacement error $\delta_{x}/{∆x}_{ref\;}$ averaged over the entire fluid domain, as shown in Figure 10. Moreover, normalized estimated displacement errors of smart-PIV measurements at lower $Re_{inflow}$ (cases $Re_{inflow}$ = 43 and $Re_{inflow}$ = 85) became comparable with conventional PIV normalized estimated displacement errors.

**FIGURE 11:**
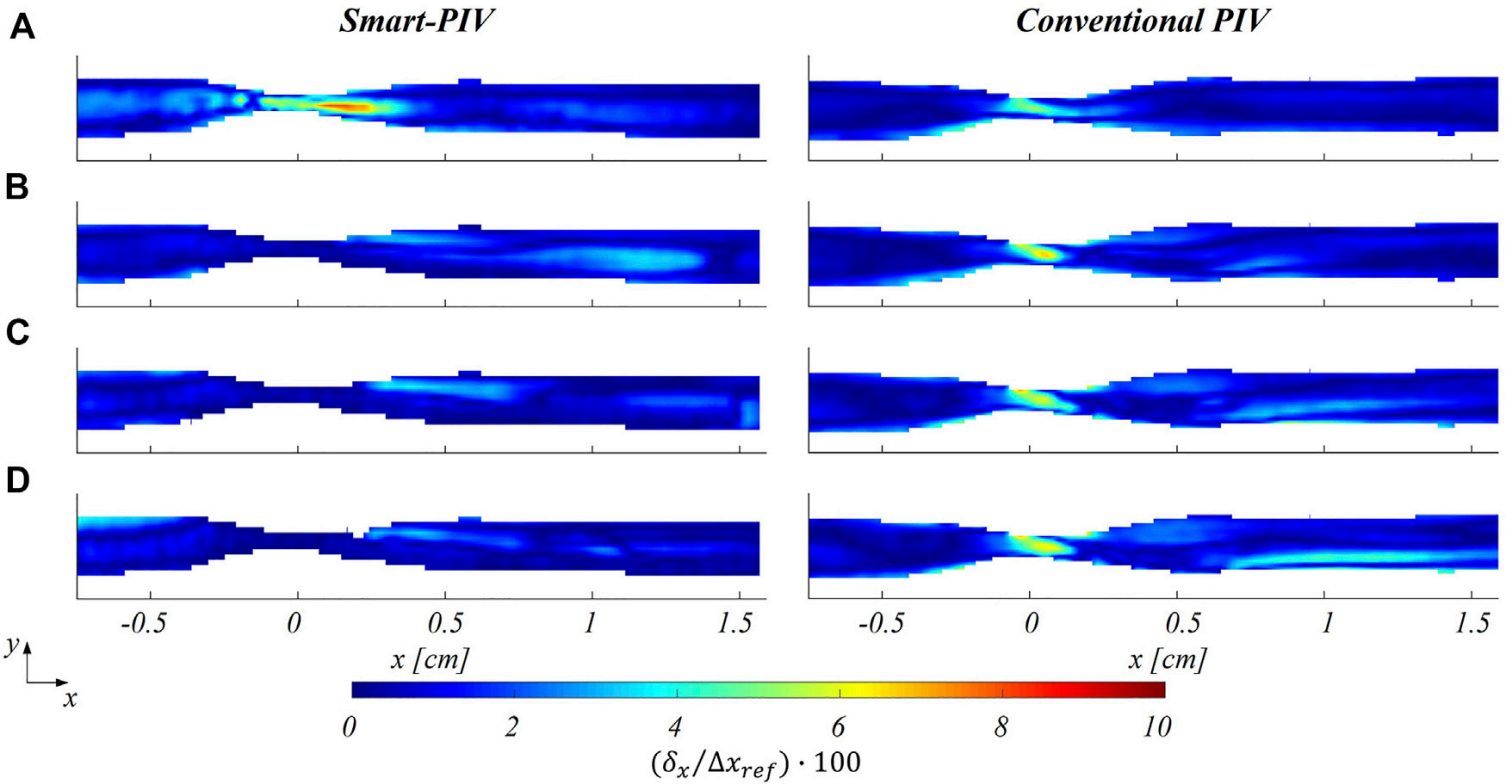
Color maps of smart (left panel) and conventional (right panel) PIV displacement error along the streamwise direction $\delta_{x}$ , normalized to the maximum streamwise particle displacement ( $∆x_{ref}$ ) at four different flow regimes: **(A)** $Re_{inflow}$ = 21; **(B)** $Re_{inflow}$ = 64; **(C)** $Re_{inflow}$ = 107; **(D)** $Re_{inflow}$ = 171 for the stenosed LAD phantom.

**FIGURE 12:**
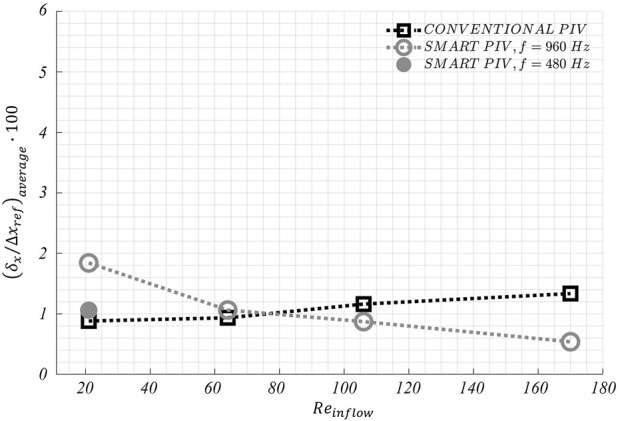
Variations of the average displacement error along the streamwise direction $\left(\delta_{x}\right.$ ) normalized by maximum streamwise particle displacement ( $∆x_{ref}$ ) as a function of the inflow Reynolds number ( $Re_{inflow}$ ) in the stenosed LAD phantom. For smart-PIV, the normalized average displacement error $\delta_{x}/∆x_{ref}$ is evaluated also by virtually reducing the image acquisition frequency to 480 Hz (for $Re_{inflow}$ = 21).

The distribution of normalized estimated displacement errors along the streamwise direction in the stenosed LAD phantom is reported in Figure 11. Smart-PIV measurements presented normalized estimated displacement errors lower than conventional PIV, with the only exception of $Re_{inflow}$ = 21 for which the normalized estimated displacement errors were comparable. Moreover, in conventional PIV measurements the highest values of $\delta_{x}/{∆x}_{ref\;}$ were mainly located within the stenosis region at all investigated flow regimes, a consequence of the expected velocity gradient in the streamwise direction generated by the lumen area reduction. Notably, the absolute estimated displacement error $\delta_{x}$ values were around 0.4 pixels, while the maximum displacement was set to be in the range 8−10 pixels. The lower values of $\delta_{x}/{∆x}_{ref\;}$ in smart-PIV measurements in the stenosis region with respect to conventional PIV measurements appear to be in contradiction with the underestimation of the stenotic peak velocity reported in Figure 8. An explanation for this only apparently contradictory result is in the fact that the uncertainty generated by particles blurring in strong gradient regions of the fluid domain using smart-PIV cannot be accounted for by the present *a posteriori* uncertainty quantification approach but can only be ascertained through comparison with conventional PIV. Relatively high normalized estimated displacement errors affecting conventional PIV measurements can also be observed in the recirculation region (Figure 11), due to an out-of-plane motion generated by the separated flow (Peng et al., 2016; Freidoonimehr et al., 2021b). Similarly, the post-stenotic jet measurements were associated with an increment of $\delta_{x}/{∆x}_{ref\;}$ at x ≈ 0.6 mm (Figure 11), where the realistic 3D geometry of the phantom and the jet flow are expected to generate local out-of-plane motion (Ding et al., 2021). The contribution of the out-of-plane motion to the normalized estimated displacement errors is influenced by the thickness of the laser sheet (≈1 mm for both systems), which was smaller than the fluid domain length scale (inlet diameter $d_{a}$ ≈ 3 mm). To complete the analysis on the stenosed LAD phantom, the normalized estimated displacement error $\delta_{x}/{∆x}_{ref}$ averaged over the entire fluid domain is presented in Figure 12. Also in this case, similar average values of $\delta_{x}/{∆x}_{ref}$ on the fluid domain under investigation were obtained between smart-PIV and conventional PIV, except for the flow regime characterized by $Re_{inflow}$ = 21. For that case, a virtual reduction of the image acquisition frame rate of the smartphone camera to 480 Hz reduced the normalized estimated displacement error $\delta_{x}/{∆x}_{ref}$ averaged over the entire fluid domain (Figure 12).

In addition, the uncertainty affecting particle displacements $U_{∆x_{i}}$ along the streamwise direction in the healthy and stenotic LAD phantoms reached maximum values below 1.2 pixel for both smart and conventional PIV.

The analysis of the normalized estimated displacement errors for the spanwise velocity component is reported in Supplementary Figures S10, S11. Normalized estimated displacement errors exhibit similar distribution as for the streamwise velocity, but they reach higher values, mainly due to the small displacements occurring along this flow direction for both healthy and stenosed LAD phantoms.

## 4 Discussion

### 4.1 Summary and implications of the findings

The developed smart-PIV set-up successfully lowered the barriers of PIV measurements in cardiovascular applications in terms of energy consumption, costs, maintenance, and safety. Leveraging cameras embedded inside commercial smartphones and low-power light sources, 2D PIV measurements were previously performed on a free water jet by Cierpka et al., 2016 recording images at 240 Hz with a 1280 × 720 pixels resolution: all factors limiting the application to moderate flow velocities and coarse spatial resolutions. Here we demonstrated that PIV measurements performed with a test bench adopting cameras embedded inside commercial smartphones and low-power *cw* light sources can be successfully extended to cardiovascular applications. The proposed set-up decreased drastically the hardware investment from roughly one hundred thousand euros of the conventional PIV set-up, where high speed cameras, a high-energy laser source and a synchronization unit were adopted, to a few thousand euros for the smart-PIV. As a further advantage, the *cw* laser used in the smart-PIV system is safer and less hazardous than pulsed lasers of conventional PIV systems, requiring less precautions to be adopted for its use as it belongs to Class 3B according to the classification of the international standard IEC 60825-1. These advantages make the smart-PIV approach more portable and usable in a wider context, enabling its use for low-cost and practical investigations for educational, industrial and research purposes. Moreover, it may prove useful as a first-line investigation, to direct and guide subsequent conventional PIV measurements.

The findings of this study proved the ability of smart-PIV technique in capturing the main coronary flow features, such as stenotic jets and post-stenotic recirculation regions (Figure 5 and Figure 8). The performance of the proposed approach, its requirements and range of applicability were defined and evaluated against conventional PIV measurements. Smartphone cameras with image acquisition frequency of 960 Hz were able to provide qualitative flow pattern visualizations and quantitative 2D velocity vector fields in realistic coronary artery phantoms in substantial agreement with conventional PIV measurements.

The normalized estimated displacement errors affecting smart-PIV and conventional PIV measurements, evaluated with the particle disparity method, were comparable at the flow regime with the highest $Re_{inflow}$ investigated (Figure 9 and Figure 11). Conversely, at the lowest inflow regimes smart-PIV measurements presented normalized estimated displacement errors higher than conventional PIV, a consequence of the fixed image acquisition frame rate of the smartphone camera. However, this limitation of the smart-PIV system could be easily circumvented by virtually reducing the image acquisition frame rate before applying the ensemble correlation: this operation had the effect of increasing the particles displacement between consecutive frames, leading to a reduction of the normalized estimated displacement errors at the lowest flow regimes (Figure 10 and Figure 12). Moreover, the uncertainty affecting particle displacements was below 1.2 pixels for smart and conventional PIV in both phantoms. This is a further confirmation that smart-PIV can be an effective alternative to conventional PIV, given a careful a priori consideration of the investigated flow regimes.

### 4.2 Current technical constraints of smart-PIV set-up and future outlook

Our findings suggest that two main technical constraints must be taken into account when planning smart-PIV measurements.

First, the maximum magnification of the field of view is fixed, impacting the size of the interrogation area and the size of the flow structures to be resolved. Because of the fixed focal length lenses embedded in commercial smartphones, the only way to increase the magnification *M* of the field of view when higher resolutions are needed is by reducing the distance between the smartphone camera and the measurement plane, until the out-of-focus limit. In comparing the performance of smart-PIV *vs.* conventional PIV, it should be mentioned that even though in the former the magnification *M* can be more than one order of magnitude smaller than the one usually encountered in the latter, the final image resolution was comparable for both PIV systems, due to the larger pixel size in the conventional PIV camera, ranging from 5 to 10 µm (Table 3).

Second, the combination of the (fixed) maximum acquisition frame rate of the smartphone camera and the use of a continuous light source resulted in particle blurring in correspondence of the stenosis (Supplementary Figures S4, S7), with the consequence of underestimating local velocity values (Figure 8) starting from 40 cm/s. To reduce particle blurring, a possibility could be offered by the adoption of a *cw* laser pulsed by a frequency generator, as suggested by Cierpka et al., 2021, or a pulsed low-power light source (Aguirre-Pablo et al., 2017; Käufer et al., 2021; Minichiello et al., 2021) to illuminate the image sensor for a short time, although this solution would require a synchronization unit. Particle blurring (Oh et al., 2021) could potentially be reduced also by decreasing the exposure time. Although it was not possible to manually adjust the exposure time in the “super slow modality” of the smartphone adopted in this study, it is expected it will become an available option in the near future, possibly through the adoption of specific smartphone apps. In addition, the rapid speed up in smartphone cameras technologies has recently pushed the camera image acquisition rate at 1920 Hz (e.g., Xiaomi 12 Pro, Huawei Mate 40 Pro). This technical improvement by itself is expected to positively impact the quality of the smart-PIV measurements, minimizing gradients effect and consequently reducing the noise affecting the measurements. Moreover, this would expand the range of applicability of smart-PIV measurements to flow fields characterized by high velocity (Figure 1). In this sense, in the last 6 years the image acquisition frame rate of smartphone cameras increased by a factor 8 (Cierpka et al., 2016), thus giving the possibility of scaling down of the same factor the minimum measurable displacement for a given flow velocity magnitude.

The impact of the discussed technical constraints on the measurements of high flow velocity could be mitigated by adopting scaled-up phantoms in the smart-PIV approach. As reported in Table 1, this is a common solution in the design of *in vitro* experiments in coronary arteries, with the practical advantage of decreasing the fluid velocity to be measured by virtue of the fluid dynamics similitude. As an example, we report here that realizing a stenosed coronary artery phantom in a scale 3:1 will result in peak velocities within the stenosis of 30 cm/s for the case at higher flow regime ( $Re_{inflow}$ = 171).

To sum up, these current technical constraints of the smart-PIV set-up should be accurately assessed to determine the flow velocity range that can be investigated and establish a priori the applicability of the smart-PIV approach, in relation to its context of use (qualitative or quantitative cardiovascular flow visualizations). Nevertheless, the findings of the present study and the current scenario in terms of expected technological development serve as a stimulus for further adoption of the smart-PIV approach in a larger variety of cardiovascular applications in the very near future. In this sense, the here adopted ensemble correlation method (Santiago et al., 1998) for velocity vector field measurement and particle disparity method (Sciacchitano et al., 2013) for the estimation of particle displacement errors and the related uncertainty has proven to be appropriate for smart-PIV applications to the characterization of steady cardiovascular flows.

### 4.3 Limitations

The main limitation of the developed set-up regards the fact that the shutter speed (and thus exposure time) cannot be adjusted in the “super slow motion” modality adopted here. Moreover, systematic errors can be caused by the rolling shutter of the smartphone camera, especially in regions of high velocity (Käufer et al., 2021).

## 5 Conclusion

This study explores for the first time the feasibility of smart-PIV measurements for the *in vitro* characterization of cardiovascular flows, with a focus on coronary flows. The sustainable, easy-to-manage, safe and low-cost proposed solution allows to perform qualitative and quantitative flow measurements for biomedical applications. The limited maximum image acquisition frame rate of smartphone cameras should be considered a priori to assess the applicability of the smart-PIV approach. However, the speed up in the evolution of smartphones technology is expected to overcome such limitations in the very near future, promoting a growing use of smart-PIV measurements for research, educational, and industrial purposes.

## Data Availability

The raw data supporting the conclusions of this article will be made available by the authors, without undue reservation.
