# Supplementary material for: Smartphone-based particle image velocimetry for cardiovascular flows applications: A focus on coronary arteries
Source: Front Bioeng Biotechnol. 2022 Dec 8;10:1011806. doi: 10.3389/fbioe.2022.1011806 (PMC9772456; doi:10.3389/fbioe.2022.1011806)
Supplement: Supplementary file 1 [file DataSheet1.PDF]

Supplementary Material

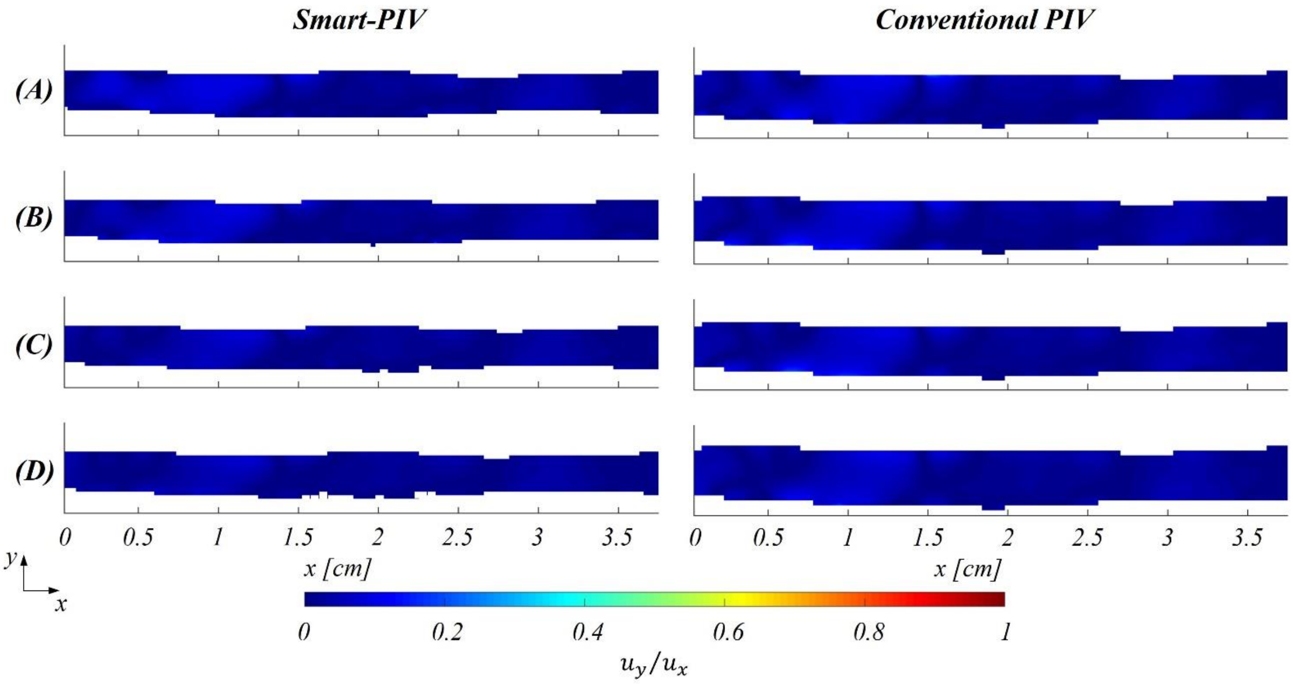

Supplementary Figure 1. Contour map of the ratio between the spanwise velocity component (u_y) and the streamwise component (u_x) for smart (left panel) and conventional (right panel) PIV at four different flow regimes: (*A*) Re_{inflow} = 43, (*B*) Re_{inflow} = 85, (*C*) Re_{inflow} = 171, (*D*) Re_{inflow} = 213 for the healthy left anterior descending (LAD) coronary artery.

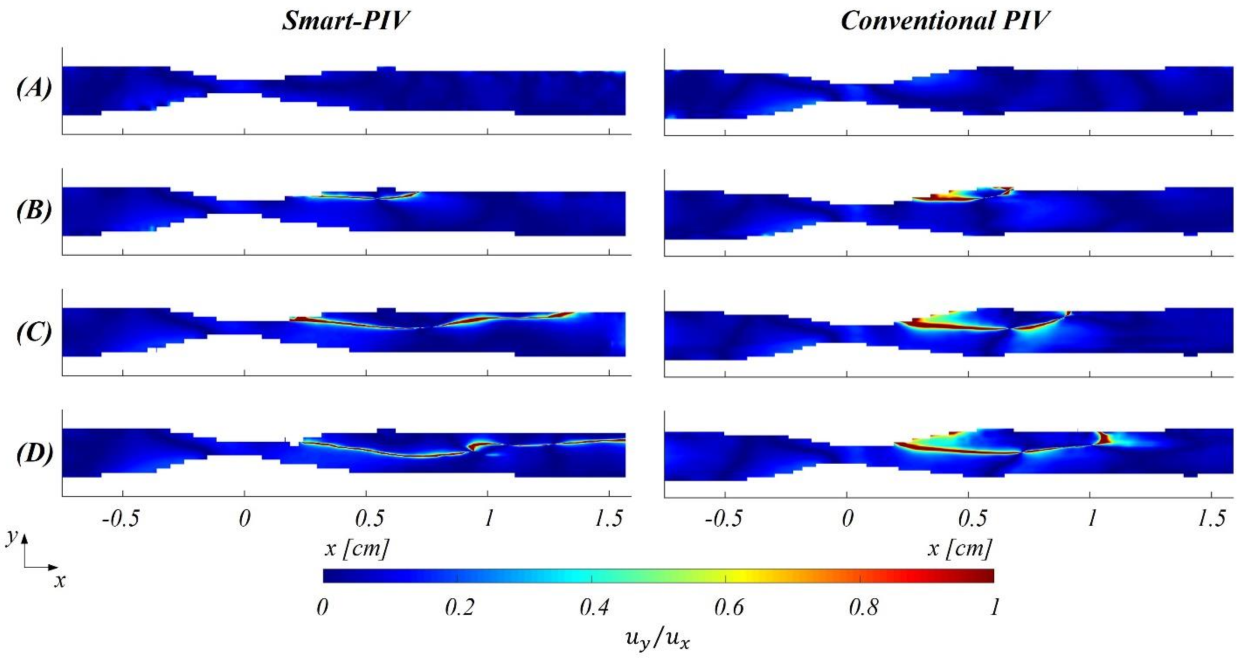

Supplementary Figure 2. Contour map of the ratio between the spanwise velocity component (u_y) and the streamwise component (u_x) for smart (left panel) and conventional (right panel) PIV at four different flow regimes: (*A*) Re_{inflow} = 21, (*B*) Re_{inflow} = 64, (*C*) Re_{inflow} = 107, (*D*) Re_{inflow} = 171 for the stenosed LAD.

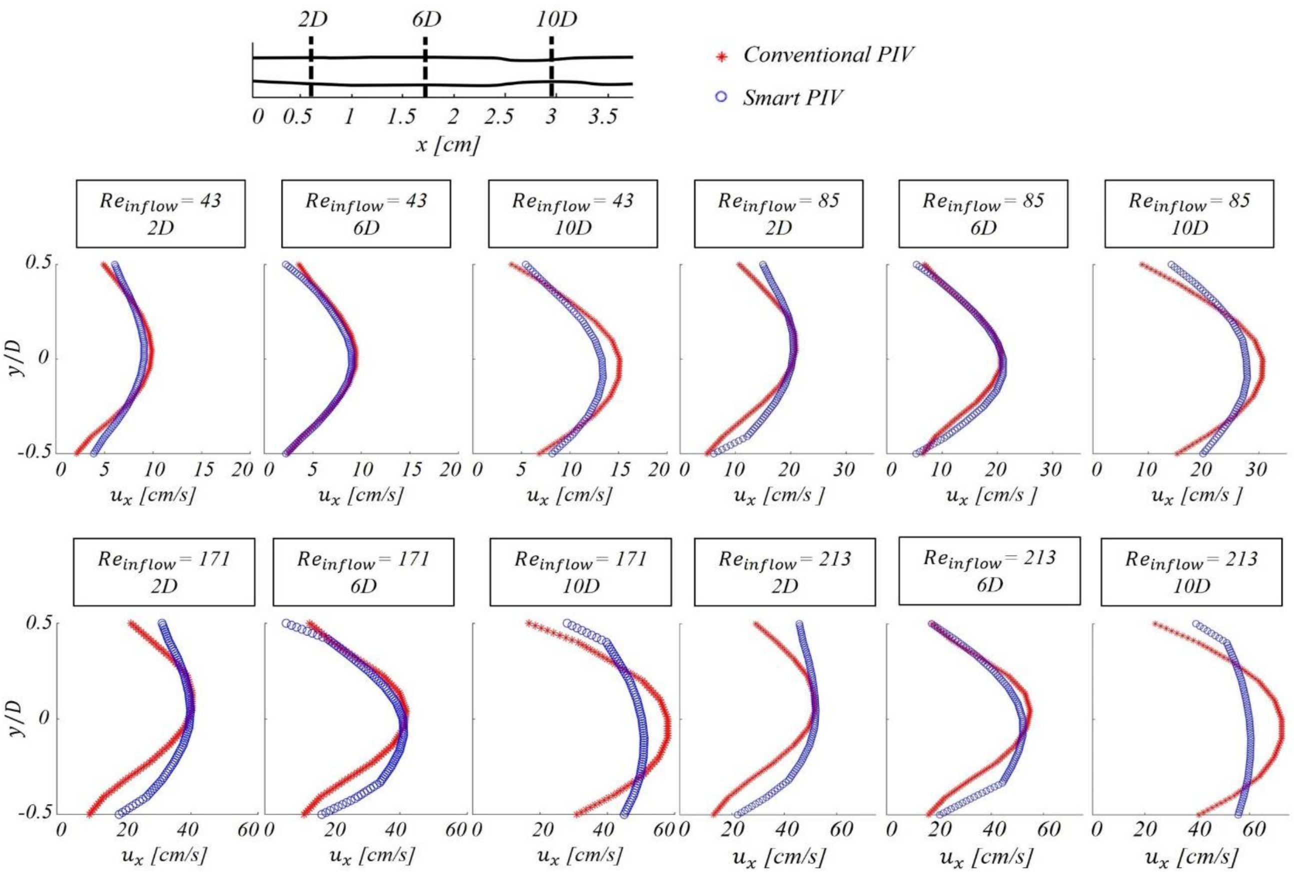

Supplementary Figure 3. Smart and conventional PIV streamwise velocity profiles for the healthy phantom at the investigated Reynolds number. The profiles were extracted at three sections, whose distance from the inlet is indicated in terms of inlet diameter *D*.

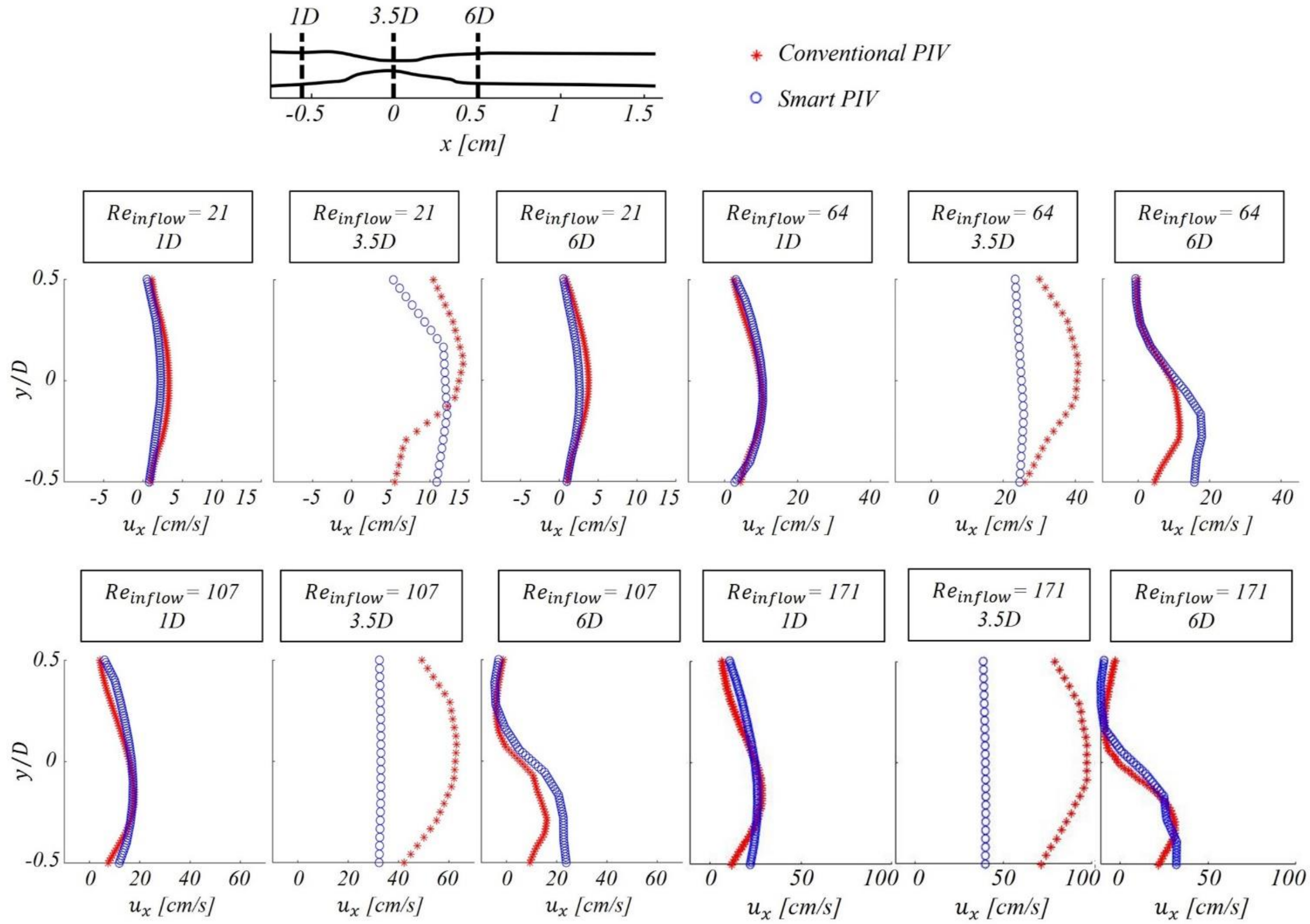

Supplementary Figure 4. Smart and conventional PIV streamwise velocity profiles for the stenosed phantom at the investigated Reynolds number. The profiles were extracted at three sections, whose distance from the inlet is indicated in terms of inlet diameter *D*.

To complete the uncertainty analysis based on particle displacements, a quantitative comparison between the velocity profiles obtained with the smart and conventional PIV at different locations was performed in terms of average absolute difference (Δ_A) and average percentage difference (Δ_B). The former was performed according to the following equation:

$$\Delta_A = \frac{1}{N}\sum_{i=1}^{N}\left|u_{x,i}^{Smart} - u_{x,i}^{Conventional}\right|$$

where $u_{x,i}^{Smart}$ and $u_{x,i}^{Conventional}$ are the smart and conventional PIV velocity profiles, respectively, and N is the total number of data points per velocity profile.

The latter was computed as follows (Bonfanti et al., 2020):

$$\Delta_B = \frac{1}{N}\sum_{i=1}^{N}\frac{\left|u_{x,i}^{Smart} - u_{x,i}^{Conventional}\right|}{max_j\ (\left|u_{x,j}^{Conventional}\right|)}$$

where $u_{x,i}^{Smart}$ and $u_{x,i}^{Conventional}$ are the smart and conventional velocity profiles, respectively, N is the total number of data points per velocity profile and j is the j-th velocity profile.

The obtained results are reported in Supplementary Figures 5 and 6. It is possible to observe that differences in the velocity profiles as given by both Δ_A and Δ_B are consistent with the velocity profiles shown in Supplementary Figures 3 and 4. Average absolute differences are bounded below 10 cm/s in the healthy coronary for all investigated flow regimes. In the stenosed phantom, average absolute differences higher than 10 cm/s are found in the section corresponding to the stenosis (Supplementary Figure 5), where Δ_A increases significantly with the inflow Reynolds number (Re_{inflow}) as a consequence of the discussed effects of particle image blurring. Average percentage differences reported in Supplementary Figure 6 are below 10% for the healthy coronary, while they reach 50.6% in the stenosed phantom in correspondence of the stenosis at the higher investigated Re_{inflow}.

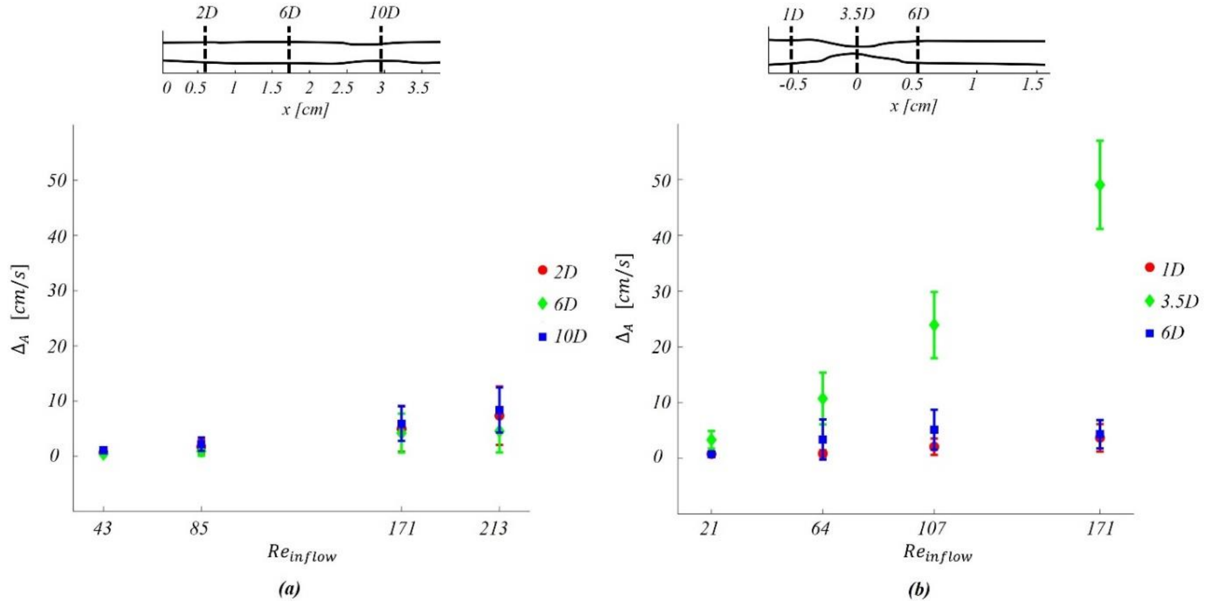

Supplementary Figure 5. Average absolute differences (Δ_A) between streamwise velocity profiles obtained with smart-PIV and conventional PIV for the healthy (panel a) and stenosed (panel b) phantoms at the investigated Reynolds number. The profiles were extracted at three sections, whose distance from the inlet is indicated in terms of inlet diameter *D*.

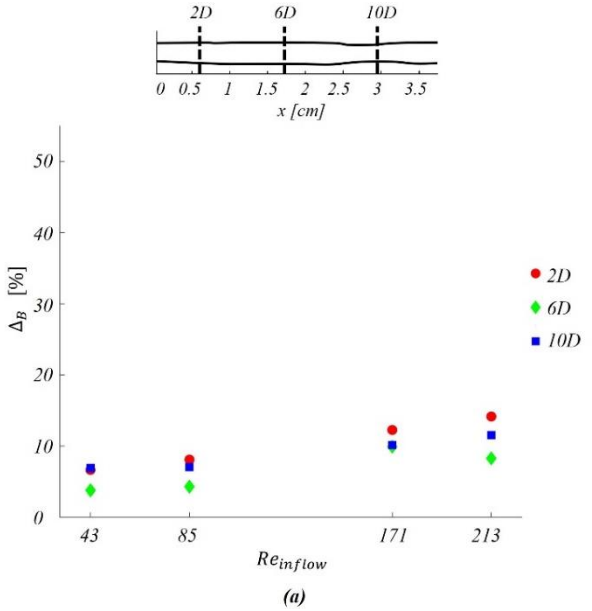

Supplementary Figure 6. Average percentage differences (Δ_B) between axial velocity profiles obtained with smart-PIV and conventional PIV for the healthy (panel a) and stenosed (panel b) phantoms at the investigated Reynolds number. The profiles were extracted at three sections, whose distance from the inlet is indicated in terms of inlet diameter *D*.

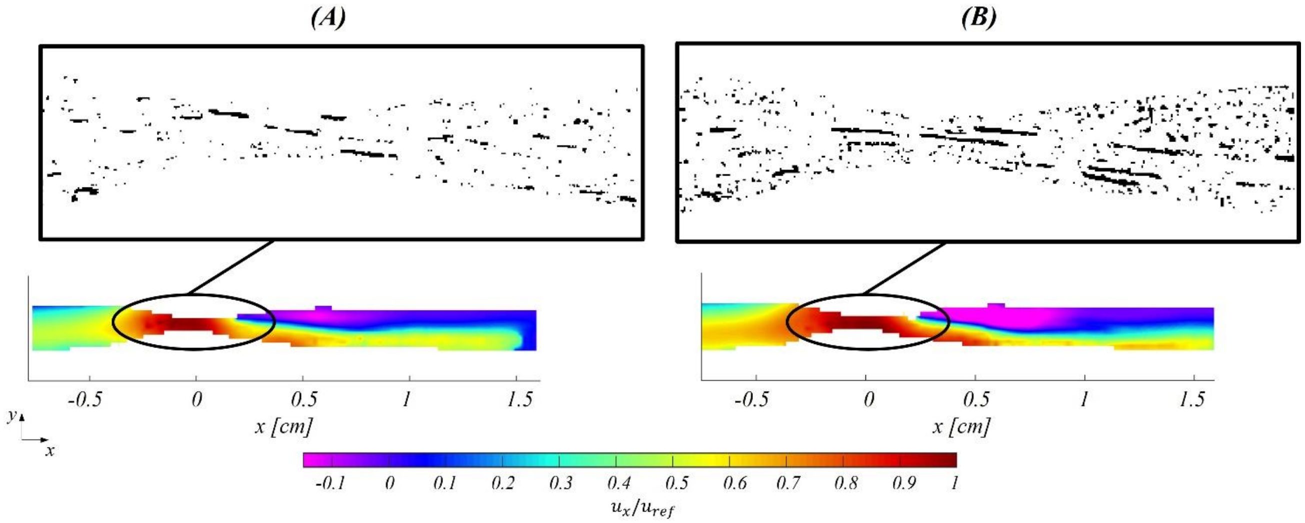

Supplementary Figure 7. Effects of particle image blur in the stenosed LAD at inflow Reynolds number equal to (*A*) 107 and (*B*) 171.

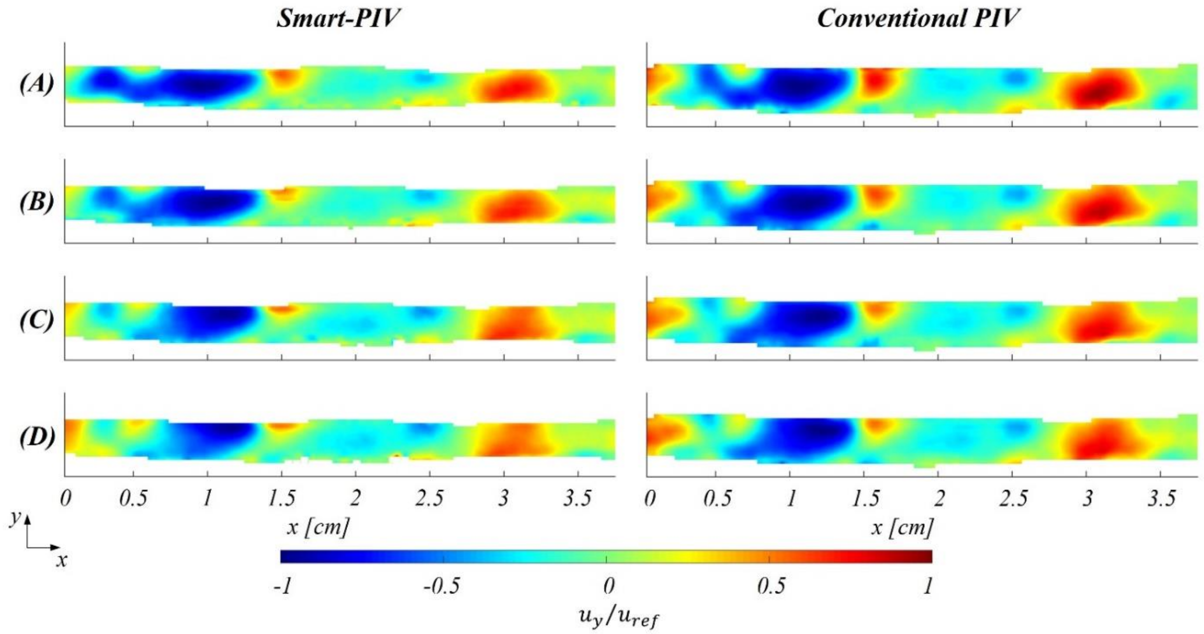

Supplementary Figure 8. Comparison of the normalized mean spanwise velocity contours between smart (left panel) and conventional (right panel) PIV at four different flow regimes: (*A*) Re_{inflow} = 43, (*B*) Re_{inflow} = 85, (*C*) Re_{inflow} = 171, (D) Re_{inflow} = 213 for the healthy LAD. The mean span-wise velocity (u_y) is normalized to the maximum spanwise velocity (u_{ref}).

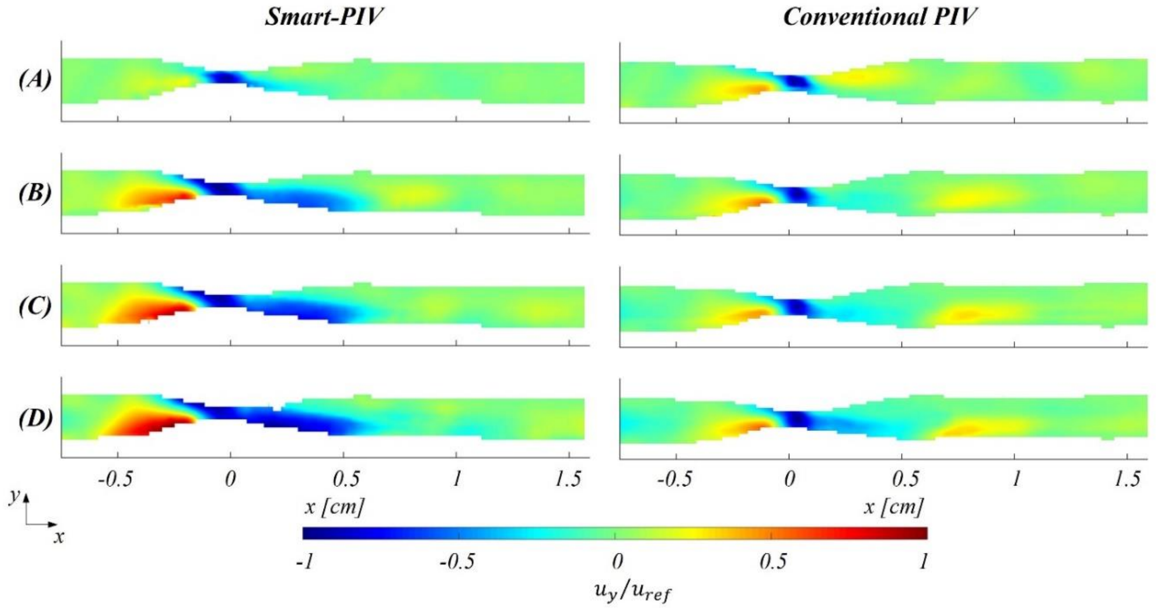

Supplementary Figure 9. Comparison of the normalized mean spanwise velocity contours between smart (left panel) and conventional (right panel) PIV at four different flow regimes: (*A*) Re_{inflow} = 21, (*B*) Re_{inflow} = 64, (*C*) Re_{inflow} = 107, (*D*) Re_{inflow} = 171 for the stenosed LAD. The mean span-wise velocity (u_y) is normalized to the maximum spanwise velocity (u_{ref}).

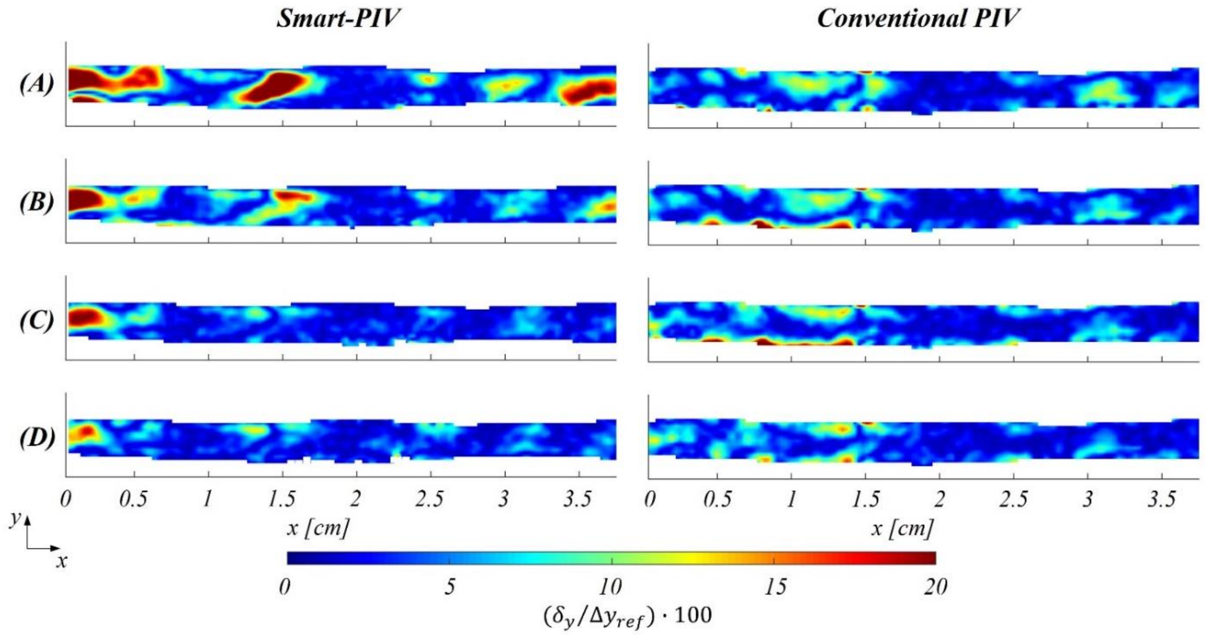

Supplementary Figure 10. Color maps of smart (left panel) and conventional (right panel) PIV spanwise error values δ_y, normalized to the maximum displacement (Δy_{ref}) at four different flow regimes: (*A*) Re_{inflow} = 43, (*B*) Re_{inflow} = 85, (*C*) Re_{inflow} = 171, (*D*) Re_{inflow} = 213 for the healthy LAD.

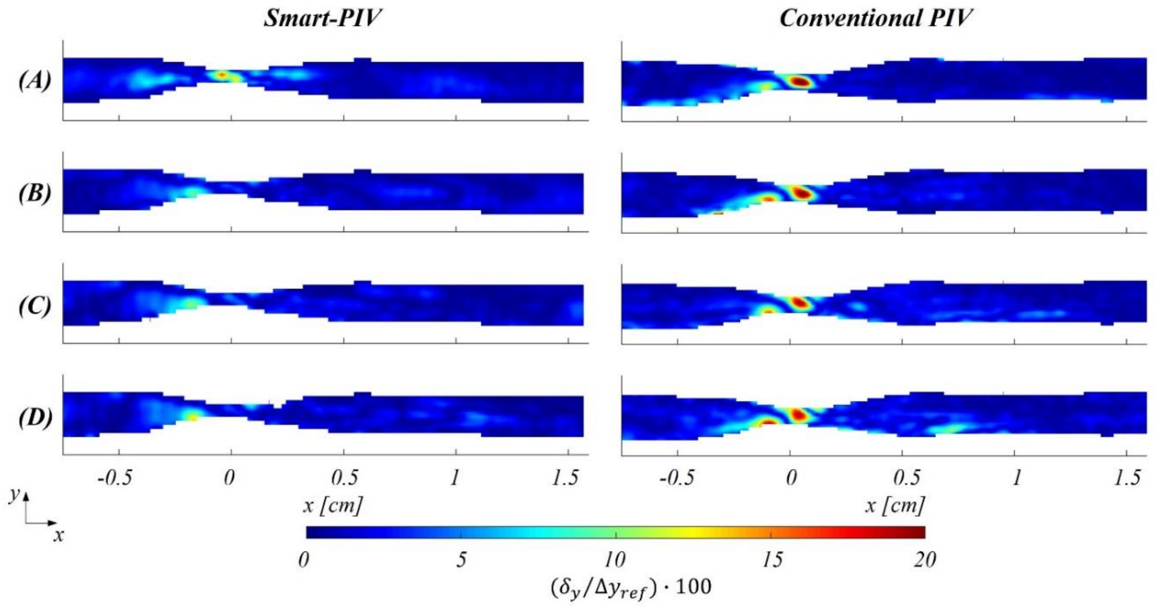

Supplementary Figure 11. Color maps of smart (left panel) and conventional (right panel) PIV spanwise error values δ_y, normalized to the maximum displacement (Δy_{ref}) at four different flow regimes: (*A*) Re_{inflow} = 21, (*B*) Re_{inflow} = 64, (*C*) Re_{inflow} = 107, (*D*) Re_{inflow} = 171 for the stenosed LAD.

Supplementary Reference

Bonfanti, M., Franzetti, G., Vanniasinkam, S.H., Dìaz-Zuccarini, V., and Balabani, S. (2020). A Combined In Vivo, In Vitro, In Silico Approach for Patient-Specific Haemodynamic Studies of Aortic Dissection. Annals of Biomedical Engineering, 48, 2950–2964. doi: 10.1007/s10439-020-02603-z
